# Assessing drinking water treatment efficiency using passive sampling and cell-based bioassays

**DOI:** 10.3389/ftox.2026.1782869

**Published:** 2026-04-23

**Authors:** Martin Ezechiáš, Michaela Hegrová Helusová, Gabriela Horáková, Eva Cséfalvay, Jaroslav Semerád, Ivana Kopecká, Tomáš Cajthaml

**Affiliations:** 1 Institute of Microbiology of the Czech Academy of Sciences, Prague, Czechia; 2 Institute for Environmental Studies, Faculty of Science, Charles University, Prague, Czechia

**Keywords:** bioassays, CYP1A, drinking water treatment, estrogenicity, gene expression, passive sampling, POCIS

## Abstract

**Introduction:**

Drinking water treatment plants (DWTPs) are designed to protect public health; however, residual contaminants may persist after treatment and elicit biological effects that are not fully covered by routine chemical monitoring.

**Methods:**

In this study, we combined Polar Organic Chemical Integrative Samplers (POCIS) with in vitro bioassays to evaluate residual biological activity in raw and treated water from six full-scale DWTPs in the Czech Republic. POCIS were deployed at raw- and treated-water points to collect extracts representing mixtures of polar and semi-polar contaminants under realistic exposure conditions. These extracts were evaluated using transcriptional responses in the RTL-W1 rainbow trout cell line and receptor-mediated yeast bioassays specific for estrogenic and progestogenic activity.

**Results:**

Among the evaluated biomarkers, gene expression of CYP1A was the most strongly and reliably induced, indicating the presence of AhR-active substances in raw water and, in several cases, incomplete removal of these substances during treatment. In contrast, genes related to phase II detoxification, cell stress, and oxidative stress (GST, HSP, and Nrf2) responded weakly, suggesting a predominantly receptor-mediated mechanism of action rather than generalized cytotoxicity. Estrogenic activity was detected in all raw waters but was below detection limits in all treated waters, indicating efficient removal of estrogenic substances during treatment. Progestogenic activity was not detectable.

**Discussion:**

This study highlights the importance of effect-directed analysis for assessing the efficiency of drinking water treatment processes and confirms the suitability of passive sampling combined with bioassays for identifying treatment-resistant bioactivities.

## 1 Introduction

Drinking water treatment plants (DWTPs) are critical infrastructure for providing safe drinking water and protecting public health by removing or reducing contaminants from raw water sources. Nevertheless, pollutants originating from industrial activity, agricultural runoff, and wastewater effluent can challenge treatment processes, and treated drinking water may still elicit harmful effects such as chronic toxicity, endocrine disruption, and adaptive stress responses (Qiao et al., 2011; Lundqvist et al., 2024; Yang et al., 2017; Neale et al., 2020). Although these adverse effects are typically associated with anthropogenic contaminants, natural substances present in raw waters can also influence treatment efficiency and biological responses. Geogenic constituents, such as arsenic, are relevant primarily due to their well-documented chronic toxicity in drinking water (Kazemifar et al., 2017). In addition, naturally occurring organic matter remains a key driver of treatment complexity, as it reacts with disinfectants, together with inorganic ions such as bromide or iodide, to form disinfection byproducts (DBPs). These DBPs are frequently linked to oxidative stress and other adverse effects observed in treated drinking water (Xiao et al., 2017; Hebert et al., 2018; Neale et al., 2020; Allen et al., 2022). Some contaminants and their transformation products can persist after treatment or act in mixtures, adding to the overall toxicological burden of drinking water and reinforcing the need to consider cumulative effects rather than individual substances alone (Smalling et al., 2023; Neale et al., 2022). This raises concerns that standard chemical monitoring may underestimate health risks, as many contaminants remain unidentified or unregulated and may exert additive or even synergistic effects. For these reasons, routine assessment should focus not only on chemical occurrence but also on the integrated toxicity of water, using effect-based monitoring and bioassays in both raw and treated water, to determine whether treatment processes effectively reduce biological hazards.

In this context, passive sampling techniques have gained increasing attention as complementary tools for monitoring trace organic contaminants. Unlike conventional grab sampling, which provides only a snapshot of water quality, passive samplers accumulate pollutants over time, enabling time-weighted average concentrations and improving detection of low-level, episodic, or transient contamination events (Vrana et al., 2005; Booij et al., 2016). Among these devices, the Polar Organic Chemical Integrative Samplers (POCIS) have emerged as a promising approach for monitoring polar and semi-polar contaminants, such as pesticides, pharmaceuticals, endocrine disruptors, and per- and polyfluoroalkyl substances (PFAS) (Harman et al., 2012; Rujiralai et al., 2011). POCIS not only preconcentrate a broad suite of chemicals but also produce extracts that are directly suitable for bioanalytical testing, facilitating integrated chemical–toxicological assessment (Alvarez et al., 2004; Neale and Escher, 2019). Recent studies have demonstrated the utility of POCIS in drinking water systems for a diverse range of emerging contaminants (Metcalfe et al., 2014; Sultana et al., 2018; Gobelius et al., 2019; Johannessen and Metcalfe, 2022), reinforcing the value of POCIS as a tool that captures realistic exposure scenarios.

When combined with *in vitro* bioassays, POCIS-based monitoring enables a powerful approach to evaluate not just chemical presence but also biological activity. Bioassays provide information on receptor-mediated effects, oxidative stress responses, and other pathways of toxicological relevance, and when applied to POCIS extracts, they offer time-integrated, mixture-relevant measures of potential hazard (Escher et al., 2018; Jálová et al., 2013; Liscio et al., 2009; Neale et al., 2022; Neale and Escher, 2019). This integrated strategy has been increasingly recognized as a promising framework for environmental toxicology, bridging the gap between chemical occurrence and health-relevant outcomes.

This study aimed to assess the effectiveness of full-scale drinking water treatment plants in reducing biologically relevant contamination in real-world source waters by coupling time-integrated passive sampling with complementary *in vitro* bioassays. POCIS were deployed at both raw- and treated-water points at six Czech DWTPs to generate time-integrated extracts representing mixtures of polar and semi-polar contaminants under realistic exposure conditions, which were subsequently tested for: cellular stress and xenobiotic-response signaling via transcript changes in RTL-W1 cells (*CYP1A*, *GST*, *HSP*, *Nrf2*) and endocrine activity (estrogenic and progestogenic) using recombinant yeast reporter assays. By comparing effect profiles before and after treatment across plants with different process configurations, we aimed to detect treatment-resistant biological activity and compare the efficacy of individual DWTPs in reducing biologically relevant hazards, thereby highlighting residual or treatment-derived bioactivity that may escape routine targeted monitoring.

## 2 Materials and methods

### 2.1 Sampling

Sampling was conducted at six DWTPs in the Czech Republic that source raw water from reservoirs. To preserve anonymity, each plant was assigned a numerical code (DWTPNo).

All treatment facilities followed broadly similar process configurations, beginning with coagulation/flocculation, followed by sedimentation (or flotation at one site). These steps were followed by sand filtration, adsorption onto granular activated carbon (GAC), and a final disinfection stage. In three of the plants, an ozonation step was incorporated upstream of the GAC units. The DWTPs differed mainly in filtration materials, GAC types, and the disinfection method applied (Table 1).

**TABLE 1:** DWTPs sampled in this study, their technical characterization, catchment characteristics, and pollution sources.

DWTP	Reservoir catchment (area in km^2^)	Pollution sources	Connected consumers	Max. Capacity [L·s^-1^]	Water treatment technological processes
DWTP1	Low-land, agricultural, partly forested, populated (2 460 km^2^)	Agriculture, WWTP effluents, recreation	∼50 000	200	Coagulation, sedimentation, or clarifiers Sand filtration (quartz sand + anthracite) GAC, UV, chloramination
DWTP2	Low-land, agricultural, partly forested, populated (2 210 km^2^)	Agriculture, WWTP effluents, recreation	∼80 000	200	Coagulation, sedimentation Sand filtration (quartz sand prepared with manganese oxides, 1–2 mm) GAC, UV, chloramination
DWTP3	Agricultural, partly forested, less populated (20 km^2^)	Agriculture, WWTP effluents, aquacultures (fishponds)	∼50 000	240	Coagulation, sedimentation Sand filtration (quartz sand 1–2 mm) Ozonation, GAC, chlorination
DWTP4	Agricultural, partly forested, populated (223 km^2^)	Agriculture, WWTP effluents, aquacultures (fishponds), cyanobacterial blooms	∼80 000	200	Coagulation, flotation Sand filtration (quartz sand 1–2 mm + anthracite 1–4 mm) Ozonation, GAC, UV, chloramination, chlorine dioxide
DWTP5	Agricultural, mostly forested, less populated (410 km^2^)	Agriculture, WWTP effluents	<5 000	80	Coagulation, sedimentation Sand filtration (quartz sand 1–2 mm) GAC, UV, chloramination, chlorine dioxide
DWTP6	Agricultural, mostly forested, less populated (21 km^2^)	Agriculture, aquacultures (fishponds)	∼15 000	80	Coagulation, sedimentation Sand filtration (quartz sand 1–2 mm) Ozonation, GAC, chlorination

A preliminary prescreening was conducted at two DWTPs with the largest reservoir catchment areas, utilizing monthly samples collected over a four-month period (April–July 2021). The April–July sampling period was selected to capture the warmer part of the year, when surface waters typically experience increased biological productivity. These samples are designated DWTPNo_4 (April) through DWTPNo_7 (July). This prescreening served to verify analytical performance and capture short-term temporal variability at the selected plants.

Following this step, the sampling campaign was expanded to include all six DWTPs, and the full set of samples was collected in July 2021. POCIS devices were deployed in stainless-steel sampling baskets at both the raw-water and treated-water locations at each DWTP. Three samplers were installed at each site. After a 30-day exposure period, the baskets were retrieved, and the POCIS units were placed into clean containers, transported to the laboratory, and stored at −21 °C until extraction.

### 2.2 Processing of the extracts from the passive sampler

The extraction of POCIS was performed according to a previously published method (Titov et al., 2024). Briefly, the Hydrophilic–Lipophilic Balanced (HLB) sorbent from each sampler was quantitatively transferred into a polypropylene syringe using ultrapure water, dried under vacuum in a Solid Phase Extraction (SPE) manifold, extracted with methanol, and evaporated under a stream of nitrogen. In parallel, non-exposed POCIS, which were kept in the laboratory and not subjected to environmental exposure, were processed using the same handling and extraction procedures. These non-exposed samplers served as procedural blanks, enabling the assessment of potential background contamination introduced during sample handling, transport, or extraction. The final volume of each extract was approximately 1 mL; these are referred to as the primary extracts.

For the viability and gene expression assays, 100 µL of each extract was transferred into a clean glass vial. The three extracts from the same sampling location were pooled (total volume: 300 µL) for subsequent experiments. To this pooled sample, 30 µL of DMSO (Dimethyl sulfoxide) (10% of the sample volume) was added, and the mixture was concentrated under a nitrogen stream until only the DMSO remained. This yielded the Concentrate of Passive Sampler Extract (CPSE).

For the estrogenic and progestogenic assays, 500 µL of each primary extract in methanol was transferred into a clean glass vial and evaporated under a gentle stream of nitrogen. The analytes were then redissolved in the same volume (500 µL) of 30% (v/v) DMSO. These steps effectively transferred the analytes from methanol into DMSO or 30% DMSO, which are less volatile than methanol.

### 2.3 Viability assays

For the viability assays, the rainbow trout (*Oncorhynchus mykiss*) cell line RTL-W1 was used. The cell line was routinely cultured in L15 medium supplemented with 5% fetal bovine serum (FBS) at 19 °C in an incubator (Pol-lab, Poland) without CO_2_, using 182.5 cm^2^ tissue culture flasks with sealed caps (VWR, Czech Republic). Cells were passaged once a week, and the medium was replaced twice weekly (Boháčková et al., 2023).

Prior to experimentation, cells were rinsed with Dulbecco’s Phosphate-Buffered Saline (DPBS) (Gibco; without calcium and magnesium), detached using trypsin/EDTA solution (0.02% trypsin, 0.5% EDTA in PBS), centrifuged for 5 min at 1200 rpm, and resuspended in L15 medium. Cells were counted using the Invitrogen Countess Cell Counter, seeded into 96-well plates at a density of 50,000 cells per well, and allowed to attach overnight. For exposure and rinsing (unless stated otherwise), a modified L15 medium (L15ex) was used, prepared according to Schirmer et al. (1997). L15ex has the same composition as L15 in terms of salts, galactose, and pyruvate, but lacks vitamins and amino acids.

Treatment solutions of L15ex and CPSE were prepared in advance. For the viability assays, the final concentration of DMSO in the treatment solutions was 1% or 0.5%. For the gene transcription assays, the DMSO concentration did not exceed 0.1%.

On the day following seeding, cells were rinsed, and 100 μL of treatment solution (CPSE diluted 100-, 200-, 400-, and 800-fold in L15ex) was added to each well. Control groups included untreated cells (100 μL of L15ex) and solvent controls (100 μL of 0.5% or 1% DMSO in L15ex).

Cell viability was assessed according to Dayeh et al. (2005), with modifications. A combination of three fluorescent indicators was used to assess different mechanisms of toxic action, enhance sensitivity, and evaluate overall cellular response (Galbis-Martínez et al., 2018). The dyes included.

- Alamar Blue (AB): assesses cellular metabolic activity
- Carboxyfluorescein diacetate (CFDA): measures cell membrane integrity
- Neutral Red (NR): evaluates lysosomal membrane integrity

After 24 h of exposure, the treatment medium was removed, and cells were rinsed twice with L15ex. Then, 100 μL of dye solution containing 0.625% AB and 0.4 μM CFDA in L15ex was added. The plate was incubated at room temperature for 30 min in the dark. Fluorescence was measured at excitation/emission wavelengths of 532/590 nm for AB and 485/535 nm for CFDA. Subsequently, the dye solution was removed, cells were rinsed, and 100 μL of NR solution (0.03 mg/mL in L15ex) was added. After 60 min of incubation, cells were rinsed twice, and NR was extracted using 150 μL of a solution containing 1% (v/v) glacial acetic acid in 50% (v/v) ethanol. The contents of each well were thoroughly homogenized using a pipette, and fluorescence was measured at excitation/emission wavelengths of 530/645 nm.

Due to the limited availability of CPSE, experiments were conducted only twice. In each experiment, cells were exposed in triplicate.

### 2.4 Gene expression assay

#### 2.4.1 Selection of genes and primer design

To monitor the potential toxicity of CPSE from raw and treated water, four genes of interest were selected, all of which are associated with cellular responses to chemical stress.

The gene encoding cytochrome P450 1A (*CYP1A*), a member of the cytochrome P450 family 1 enzymes, was selected due to its pivotal role as a primary AhR-responsive gene involved in phase I xenobiotic metabolism. This isoform preferentially metabolizes polycyclic aromatic hydrocarbons (PAHs) (Lewis et al., 1998; Burkina et al., 2021).

Another gene involved in xenobiotic metabolism, glutathione-S-transferase (*GST*), catalyzes the conjugation of glutathione to various xenobiotics, facilitating their elimination (Pérez-López et al., 2002; Riol et al., 2001). Upregulation of *CYP1A* and *GST* may indicate the presence of environmental pollutants (Behrens et al., 2001; Pérez-López et al., 2002).

The third selected gene, heat shock protein 70 (*HSP70*), encodes a molecular chaperone involved in protein folding. Increased expression of *HSP70* indicates cellular stress, including chemical stress induced by xenobiotics (Kilemade and Mothersill, 2001; Ma and Luo, 2020).

The final gene of interest, nuclear factor erythroid 2-related factor 2 (*Nrf2*), is a transcription factor that activates antioxidant responses, aiding in the detoxification of reactive oxygen species that contribute to oxidative stress (Kamai et al., 2022; Solano-Urrusquieta et al., 2020).

To normalize the gene expression data, reference genes with stable expression under experimental conditions were selected. Based on the literature (Ferain et al., 2018; Ma et al., 2019; Mahapatra et al., 2017; Santos et al., 2021; Shekh et al., 2017), seven candidate reference genes were evaluated for expression stability.

Primers were designed using the Primer3Plus online tool (Koressaar and Remm, 2007; Untergasser et al., 2012) and synthesized by Metabion International AG (Planegg, Germany). Two primer sets were designed for each gene, and both were tested for amplification efficiency. Primer sets with efficiencies ranging from 85% to 110% were selected.

Expression stability of candidate reference genes was assessed using four software tools: NormFinder, BestKeeper, geNorm, and RefFinder (Andersen et al., 2004; Pfaffl et al., 2004; Vandesompele et al., 2002; Xie et al., 2023). Based on consensus among these tools, the four most stable reference genes were selected: *18S rRNA*, ATP synthase subunit delta, ADP/ATP translocase, and ribosomal protein 8.

#### 2.4.2 Exposure of cells to extracts

Cells were cultured and prepared for the gene expression assays using the same procedures as described for the viability assays, with minor modifications. For this experiment, cells were seeded in six-well plates at a density of 500,000 cells per well and incubated for approximately 40 h.

The exposure solution consisted of L15 medium without FBS and 0.1% CPSE (diluted 1:1000). A solvent control containing 0.1% DMSO in L15 medium without FBS was included for each six-well plate. Before exposure, cells were rinsed with DPBS, and 3 mL of the exposure solution was added to each well. The plates were incubated at 19 °C for 24 h.

After incubation, the cells were rinsed with DPBS, and total RNA was isolated using the RNeasy Plus Mini Kit (Qiagen, Canada) following the manufacturer’s instructions. Genomic DNA contamination was removed using the TURBO DNA-free Kit (Invitrogen). RNA purity and concentration were assessed using a NanoDrop-1000 spectrophotometer (Thermo).

For reverse transcription, the iScript cDNA Synthesis Kit (Bio-Rad) was used, with 1 μg of total RNA per reaction. Gene expression was quantified by qPCR using the Luna Universal One-Step RT-qPCR Kit (New England Biolabs) on a LightCycler® 480 instrument (Roche, Switzerland). Each 5 μL reaction contained the Luna Universal qPCR mix primers (final concentration 0.125 μM) and a 40-fold diluted cDNA template. Reactions were run in duplicate on a 384-well plate.

The qPCR program started with an initial denaturation at 95 °C for 60 s, followed by 45 amplification cycles consisting of denaturation at 95 °C for 15 s and extension at 60 °C for 30 s. After the amplification cycles, a melting curve analysis was performed by gradually increasing the temperature from 55 °C to 95 °C over 60 s to confirm product specificity.

Data were analyzed using LightCycler® 480 SW 1.5.1 software. Fold induction was calculated using qBase software (Hellemans et al., 2007), based on the Ct values of reference and target genes and corresponding primer efficiencies.

All experiments were conducted in biological triplicate using cells from different passages to account for biological variability.

### 2.5 Estrogenic assay

Standardized estrogenic reporter gene assays were employed to assess estrogenic activity. This assay was first described by Routledge and Sumpter (1996). We used a modified procedure based on the method described by Dhooge et al. (2006), in which the authors prolonged the exposure of cells to achieve higher assay sensitivity.

Briefly, yeast cultures were incubated overnight at 30 °C until an absorbance of 0.6 was reached at 620 nm. Sample extracts in 30% (v/v) DMSO were used to prepare a dilution series for each sample. An aliquot of 20 µL of each dilution was transferred into a 96-well plate in triplicate, followed by the addition of 180 µL of the yeast culture. The final DMSO concentration in the yeast culture did not exceed 3%. Plates were incubated at 30 °C for 5 days, after which absorbance at 540 nm and 620 nm was measured using a plate reader. Corrected absorbance values, accounting for potential sample toxicity, were calculated according to Routledge and Sumpter (1996).

A standard curve of the natural ligand 17β-estradiol (E2) was measured on each plate. The assay detection limit corresponded to the lowest sample concentration producing a response exceeding 15% of the maximal E2 response, which represents the standard threshold for quantification in the yeast recombinant assay and corresponds approximately to 0.1 ppb estradiol equivalents (EEQ). For each sample dilution series, results were plotted, and the first dilution that reached the threshold of 15% of the maximal E2 response was used to calculate the estradiol equivalent (EEQ), defined as the concentration of E2 that produces the same response as the tested sample. EEQ values were corrected for both the dilution factor and the volume of the original extract, so the reported EEQ values correspond to the primary extracts.

### 2.6 Progestogenic assays

Two transfected strains of *Saccharomyces cerevisiae* were employed: BMA64/luc (viability control) and BMAEREluc/PR (progestogenic activity). These were used to measure the progestogenic activity of passive sampler extracts and their serial dilutions. The method was adapted from Leskinen et al. (2005) with slight modifications.

Briefly, yeast cultures were incubated overnight at 30 °C and 230 rpm in growth medium according to the original protocol. The cultures were then diluted to an optical density of OD_640_ = 0.4 and incubated until reaching OD_640_ = 0.6–0.7 under the same conditions. For the assay, 10 µL of each sample in 30% (v/v) DMSO was pipetted into a sterile white 96-well plate (TC surface-modified; SPL, Biotech a. s., Czech Republic), and 100 µL of the growing yeast culture was added. The final DMSO concentration did not exceed 3%.

After 2.5 h of incubation at 30 °C, bioluminescence was measured using a GloMAX® Discover microplate reader (Promega, Czech Republic) following the addition of 100 µL of a 0.1 M D-Luciferin solution (Duchefa Biochemie, Biotech a. s., Czech Republic) in Na-citrate buffer (pH 5). Progesterone was used as the positive control, while 30% DMSO served as the blank. Measured luminescence values were corrected for potential compound toxicity using the control strain (Leskinen et al., 2005).

All measurements were performed in triplicate and are presented as the mean ± standard deviation.

### 2.7 Statistical analysis

All data were evaluated for statistical significance using a two-sample t-test to compare treatment groups with their respective controls (procedural blanks). Prior to analysis, data were assessed for normality and homogeneity of variances to confirm the assumptions of the t-test. Results were considered statistically significant at a p-value <0.01.

Statistical analyses were performed using OriginPro 8.5 software (OriginLab Corporation, Northampton, MA, USA). All values are presented as mean ± standard deviation unless otherwise stated.

## 3 Results

### 3.1 Viability results

Each CPSE was tested for cell viability at 100-, 200-, 400-, and 800-fold dilutions. Experiments were performed twice, with each dilution measured in triplicate. Viability data are shown in Supplementary Figure S1 for the six DWTPs and in Supplementary Figures S2, S3 for the time-series samples from DWTP1 and DWTP2 (see Supplementary Material).

A significant decrease in cell viability was observed in raw water samples compared to controls (non-exposed cells) only at the 100-fold dilution; higher dilutions did not exhibit this effect. No significant decrease in viability was detected in extracts from treated water, except for DWTP5, where the 100-fold dilution exhibited significant cytotoxicity. As expected, for each DWTP, raw water extracts had a more substantial adverse effect on viability than equally diluted extracts from treated water, again except DWTP5, where treated water retained a toxic effect comparable to that of the raw water. This distinct behaviour at DWTP5 may result from limited removal efficiency of bioactive compounds or from the formation of reactive transformation or disinfection byproducts during treatment, which can sustain or enhance cytotoxicity (Neale et al., 2020; Allen et al., 2022). The viability results also informed the design of the gene expression experiments, which are typically performed at sub-lethal concentrations. Although all samples were non-toxic at an 800-fold dilution, a 1000-fold dilution was applied as a precaution.

### 3.2 Gene expression results

**FIGURE 1:**
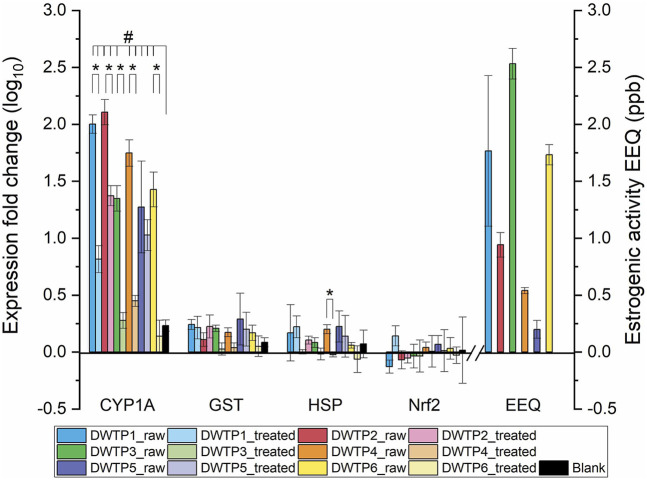
Relative expression levels (log_10_ fold change) of *CYP1A*, *GST*, *HSP*, and *Nrf2* in the RTL-W1 cell line exposed to raw and treated water from six DWTPs (DWTP1 to DWTP6). Raw water represents source water entering the DWTP, whereas treated water corresponds to finished drinking water after treatment. Black bars represent procedural blanks. Estrogenicity of raw water is expressed as estradiol equivalents (EEQ); bars for treated water are not shown because no estrogenic activity was detected. Values represent means (n = 3), with error bars standing for standard deviations. Asterisks (*) indicate statistically significant differences between raw and treated water samples (p < 0.05). Hash symbols (#) indicate statistically significant differences relative to the procedural blank (p < 0.05).

Four genes were monitored to assess cellular responses: *CYP1A* and *GST*, representing xenobiotic degradation pathways; *HSP*, a marker of general stress response; and *Nrf2*, a regulator of oxidative stress response (Figure 1). For each drinking water treatment plant (DWTP), gene expression responses were compared between raw water (source water entering the treatment process) and treated water (finished drinking water after treatment) in order to evaluate the effectiveness of the treatment processes in reducing bioactive contaminants. Among these, the strongest and most consistent expression changes were observed for *CYP1A* (Figure 1). In almost all raw and treated water extracts, *CYP1A* expression levels were significantly higher than those of the procedural blanks, demonstrating a clear induction of xenobiotic metabolism pathways by the water samples. The only exceptions were treated waters from DWTP3 and DWTP6, where *CYP1A* expression did not differ significantly from procedural blanks, indicating that advanced treatment processes (at both sites, including ozonation, GAC, and chlorination after conventional treatment) effectively reduced bioactive contaminants to levels that did not elicit measurable chemical stress responses. This outcome is also consistent with the characteristics of their reservoir catchments, as both DWTPs are supplied from relatively small and sparsely populated areas, and DWTP6 in particular receives raw water from an area without main municipal wastewater treatment inputs (Table 1). Consequently, the initial contaminant load, especially compounds capable of activating AhR signaling, is expected to be lower than in more urbanized catchments, which likely contributed to the low biological responses observed after treatment. Comparison of raw versus treated waters revealed that raw water samples consistently induced stronger CYP1A expression, with statistically significant differences observed in all DWTPs except DWTP5.

In contrast, the expression patterns of *GST*, *HSP*, and *Nrf2* remained relatively stable across treatments. None of these genes showed significant differences compared with procedural blanks (Figure 1), suggesting that pathways related to phase II detoxification, stress response, and oxidative stress were not strongly activated under the tested conditions. When comparing raw and treated waters, a significant reduction in *HSP* expression was observed only at DWTP4. This is the only significant change among our data for *GST*, *HSP*, and *Nrf2*, further highlighting *CYP1A* as the most sensitive biomarker among the genes analyzed.

**FIGURE 2:**
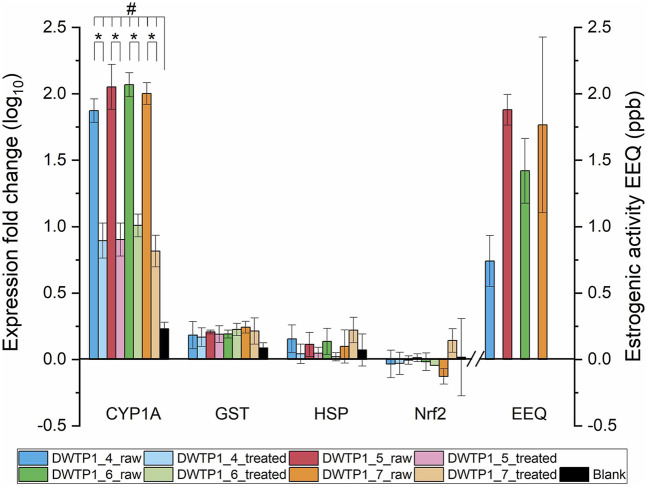
Relative expression levels (log_10_ fold change) of *CYP1A*, *GST*, *HSP*, and *Nrf2* in the RTL-W1 cell line exposed to raw and treated water from DWTP1, sampled between April and July (DWTP1_4 to DWTP1_7). Raw water represents source water entering the DWTP, whereas treated water corresponds to finished drinking water after treatment. Black bars represent procedural blanks. Estrogenicity of raw water is expressed as estradiol equivalents (EEQ); bars for treated water are not shown because no estrogenic activity was detected. Values represent means (n = 3), with error bars standing for standard deviations. Asterisks (*) indicate statistically significant differences between raw and treated water samples (p < 0.05). Hash symbols (#) indicate statistically significant differences relative to the procedural blank (p < 0.05).

**FIGURE 3:**
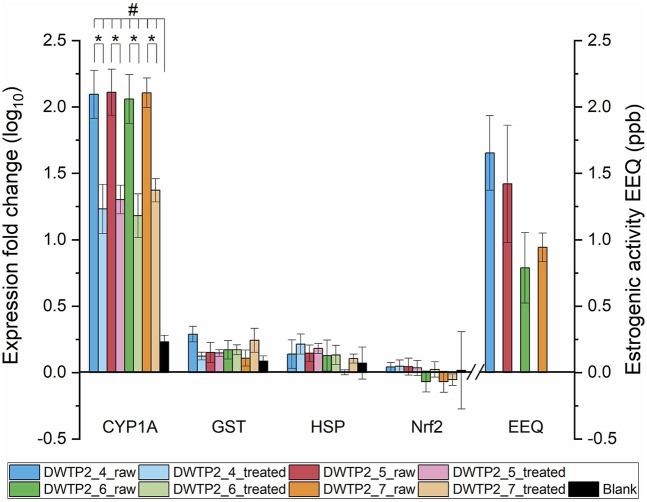
Relative expression levels (log_10_ fold change) of *CYP1A*, *GST*, *HSP*, and *Nrf2* in the RTL-W1 cell line exposed to raw and treated water from DWTP2, sampled between April and July (DWTP2_4 to DWTP2_7). Raw water represents source water entering the DWTP, whereas treated water corresponds to finished drinking water after treatment. Black bars represent procedural blanks. Estrogenicity of raw water is expressed as estradiol equivalents (EEQ); bars for treated water are not shown because no estrogenic activity was detected. Values represent means (n = 3), with error bars standing for standard deviations. Asterisks (*) indicate statistically significant differences between raw and treated water samples (p < 0.05). Hash symbols (#) indicate statistically significant differences relative to the procedural blank (p < 0.05).

Additional insight was gained from the time-series sampling at DWTP1 and DWTP2, conducted between April and July (Figures 2, 3). For both sites, *CYP1A* again exhibited the strongest expression responses. Expression levels in both raw and treated waters were consistently and significantly elevated compared with blanks, confirming the reproducibility of CYP1A induction across time. By contrast, *GST*, *HSP*, and *Nrf2* remained unchanged throughout the sampling period, reinforcing the observation that these markers were not responsive under the given exposure scenarios.

When comparing raw versus treated waters within the time series, raw water samples consistently induced higher *CYP1A* expression levels than treated water at both DWTP1 and DWTP2. This indicates that treatment processes (GAC + UV + chlorination after conventional treatment) at these facilities effectively reduced, but did not completely eliminate, the bioactive compounds responsible for *CYP1A* induction. These findings may also be related to the characteristics of the catchments supplying reservoirs for both DWTPs, as they exceed 2,000 km^2^ and encompass densely populated regions with intensive agriculture, multiple municipal wastewater treatment discharges, and significant recreational use. Such extensive and human-impacted catchments are expected to deliver a continuous and chemically complex mixture of AhR-active contaminants to the raw water, which increases the burden on downstream treatment processes and may explain the persistent *CYP1A* induction observed even after advanced treatment. Importantly, the four-month series did not reveal any consistent seasonal trend in gene expression responses. No progressive increase or decrease was observed from April through July, suggesting that the toxicological potential of both raw and treated waters remained relatively stable over the studied period.

### 3.3 Hormonal activity results

The progestogenic yeast assay demonstrated that across all DWTPs none of the tested samples exhibited detectable progestogenic activity. Given the absence of activity, data on progestogenic responses are not shown.

In contrast, pronounced effects were observed in the estrogenic yeast assay. All raw water extracts induced significant estrogenic activity, expressed as estradiol equivalents (EEQ) (Figure 1). Among the six DWTPs tested, the highest estrogenicity was detected in raw water for DWTP3, followed by DWTP1 and DWTP6, while the lowest EEQ values were observed in DWTP5. These results demonstrate that estrogenic activity was a consistent feature of raw water samples, although its magnitude varied across treatment plants.

Notably, treated waters did not display any measurable estrogenic activity in our assays. Estrogenic responses in these samples were consistently below the detection threshold (∼0.1 ppb EEQ), as was the case for procedural blanks. This pattern suggests that the treatment processes applied at the studied DWTPs were effective in removing or reducing estrogenic compounds to levels below detection.

The time-series analysis at DWTP1 and DWTP2 further confirmed these findings (Figures 2, 3). At both sites, raw water samples repeatedly exhibited significant estrogenic activity, whereas treated water samples consistently showed no detectable activity throughout the April–July sampling period. Despite monthly variation in the magnitude of EEQs in raw water, no consistent pattern was apparent across the 4 months of observation (Figures 2, 3). Thus, while raw water estrogenicity persisted over time, the treatment process reliably reduced activity to undetectable levels, with no evidence of seasonality in the measured effects.

## 4 Discussion

The most prominent transcriptional response was observed for *CYP1A*, a sensitive biomarker of exposure to polycyclic aromatic hydrocarbons (PAHs) and other related aromatic xenobiotics. In addition to PAHs, CYP1A is also strongly induced by some pesticides (Xing et al., 2014; Noor and Rahman, 2025) or by planar halogenated aromatic hydrocarbons (PHAHs) such as polychlorinated biphenyls (PCBs), which are substrates of CYP1A-mediated biotransformation and potent activators of AhR signaling (Bandiera, 2001; Thomas and Fent, 2009). It was also demonstrated that exposure to coplanar PCBs such as PCB 77 markedly increases *CYP1A* mRNA and protein levels as well as EROD (ethoxyresorufin-O-deethylase) activity in hepatic tissues of marine and freshwater teleosts, and similar induction has been observed following exposure to PCB mixtures such as Aroclor 1254 (Rahman and Thomas, 2018). Collectively, these findings indicate that *CYP1A* expression is highly susceptible to modulation by PCB contamination, as well. *CYP1A* induction was consistently higher in raw water compared with treated water, indicating that treatment processes reduced, but did not fully eliminate, compounds capable of activating the AhR pathway. This persistence of AhR activity is consistent with findings from the literature (Enault et al., 2023). Among the applied processes, the reduction of compounds capable of activating the AhR pathway is often attributable to GAC adsorption, which is generally effective in removing hydrophobic compounds such as PAHs, PCBs, and other micropollutants (Lamichhane et al., 2016; Rosińska, 2017; Eeshwarasinghe et al., 2019). Lundqvist et al. (2024) confirmed the effective removal of AhR activity using several types of GAC adsorption filters as well as biofilters, both at full-scale water treatment facilities and in a pilot-scale system. Ozonation, when implemented upstream of GAC, generally provides a more consistent and sustained reduction in bioactivity, owing to its capacity to oxidize aromatic structures prior to adsorption. Conversely, GAC filtration operated without pre-ozonation often exhibits declining performance over time and therefore requires regular regeneration to maintain its removal efficiency (Yu et al., 2021). In contrast, expression of *GST*, *HSP*, and *Nrf2* remained relatively stable across samples and did not differ from procedural blanks, indicating that these genes were not significantly induced under the tested conditions. Gene expression levels were successfully quantified in all samples, confirming that the assay was sufficiently sensitive to detect transcriptional responses. However, because the extracts were tested at a 1000× dilution, subtle biological responses cannot be entirely excluded. Overall, these results highlight *CYP1A* as the most sensitive biomarker among the genes analyzed. Similar findings have been reported in previous studies, where *CYP1A* frequently emerged as the most responsive endpoint to complex waterborne contaminant mixtures (Behrens et al., 2001; Burkina et al., 2021). The absence of variation in *CYP1A* expression across the April-July time series further suggests that the toxicological potential of these waters remained relatively stable over the short term. The April–July sampling period was selected to capture conditions when surface waters are typically subject to increased biological activity and anthropogenic pressures, including agricultural runoff, recreational use, and enhanced microbial and algal productivity. These factors may influence the occurrence and transformation of bioactive contaminants in reservoir catchments. Although the four-month time series did not reveal clear temporal trends in the measured responses, seasonal variability cannot be excluded, and sampling across a full annual cycle would provide a more comprehensive understanding of temporal dynamics in bioactive contaminant mixtures.

Our *Nrf2* expression results merit comparison with previous research. Wang et al. (2013) demonstrated that organic contaminant extracts from drinking water activated the Nrf2-mediated antioxidant response in HepG2 human liver cells, including elevated Nrf2 protein levels and induction of ARE-regulated genes, even at concentrations only modestly above environmental levels. Kamila et al. (2025) also reported robust activation of the Nrf2–Keap1–ARE pathway in zebrafish kidney tissue exposed to arsenic and chromium, demonstrating that metals and complex mixtures can effectively stimulate antioxidant defenses and DNA repair. In contrast, we observed no induction of *Nrf2* signaling. The most likely explanation for this discrepancy is the use of POCIS as a sampling tool in our study, which enriches a distinct subset of waterborne contaminants and thus produces a chemical profile that may differ substantially from the bulk water extracts used by other investigators. Nevertheless, other factors such as differences in biological model, contaminant composition, or sampling strategy may also contribute to these contrasting findings. Together, these considerations highlight how both sampling strategy and biological system influence the detection of Nrf2-mediated responses.

In contrast to the significant induction of *HSPs* observed in several field and *in vitro* studies, our RTL-W1 cell assays did not show elevated HSP expression. For example, An et al. (2014) found markedly increased *HSP30* and *HSP70* mRNA levels in the liver and kidney of wild crucian carp collected downstream of a polluted watershed, demonstrating that exposure to complex mixtures of contaminants in the field readily induces *HSP* pathways. Similarly, Muposhi et al. (2015) reported elevated *HSP70* protein levels in the stomach tissue of Nile tilapia exposed to heavy-metal-contaminated river water, further underscoring *HSP70* as a responsive biomarker to metal pollution. Another study in controlled *in vitro* models found that primary epidermal cultures from rainbow trout exhibited dose-dependent *HSP70* induction following exposure to the xenobiotic 2,4-dichloroaniline, highlighting *HSP70’s* sensitivity even in isolated cell systems (Kilemade and Mothersill, 2001). By contrast, our system, comprising RTL-W1 hepatocyte-like cells exposed to POCIS extracts, may not deliver the threshold or the chemical classes necessary to elicit proteotoxic or oxidative stress sufficient to induce *HSP* expression. Moreover, intact organisms integrate HSP signaling across tissues and organ systems over longer durations, while our acute and targeted *in vitro* exposures may not engage the same stress response dynamics. Our results suggest that *HSPs* are more informative in long-term field biomonitoring contexts than in short-term *in vitro* assays, highlighting the need to select biomarkers according to both sampling strategy and biological model.

Having characterized stress- and detoxification-related responses, we next evaluated the endocrine-disrupting potential of the water samples. Our receptor-based yeast assays did not detect any progestogenic activity in either raw or treated water samples from the six DWTPs. This finding is consistent with the literature, which indicates that progestogenic activity in drinking water is generally low or below detection limits. Broader environmental monitoring studies across multiple countries have reported that progestogenic activity is detectable in surface waters and treated wastewater, but is largely absent in drinking water samples (Leusch et al., 2018; Houtman et al., 2021; Rocha and Rocha, 2022; Šauer et al., 2018). Estrogenic activity, on the other hand, was consistently observed in raw water samples across all six DWTPs in our study. Levels varied between plants, with the highest estradiol equivalents (EEQs) measured at DWTP3 and the lowest at DWTP5, reflecting differences in source water contamination pressures. The observed estrogenic responses in all raw water samples likely stem from a complex mixture of both natural steroid hormones excreted by humans and animals (e.g., estrone, estriol, 17β-estradiol, 17α-ethinylestradiol) and synthetic or anthropogenic estrogen-mimicking chemicals (so-called xenoestrogens), such as bisphenols, alkylphenols, phthalates, or pesticide residues. These compounds may enter surface and raw waters through municipal wastewater effluents, agricultural runoff, industrial discharges, plastic and consumer product leachates, or insufficiently treated waste streams (Wu et al., 2017; Grzegorzek et al., 2024). Because conventional wastewater treatment plants often do not completely remove these substances, residual levels persist and may result in measurable estrogenicity in raw water destined for drinking water treatment. As a consequence, the measured EEQ likely reflects the cumulative estrogenic burden from diverse sources rather than a single compound, underlining the importance of comprehensive monitoring (both bioassays and chemical analyses) and, if needed, implementation of advanced removal or barrier technologies (Wang et al., 2011; Grzegorzek et al., 2024). Importantly, estrogenic activity was completely absent in all treated water extracts, with responses indistinguishable from procedural blanks. These findings indicate that, despite variability in raw water quality, the treatment processes applied were effective at removing estrogenic compounds to below detection levels. Comparable removal efficiencies have been reported in European DWTPs (König et al., 2017). Leusch et al. (2018) also reported detectable estrogenic activity in surface waters, whereas drinking water samples lacked measurable estrogenic effects. Rosenmai et al. (2018) similarly showed that ER activity was consistently present in raw and early treatment stage samples but almost entirely absent in produced drinking water. This closely aligns with our findings: estrogenic effects were detected in all raw-water samples, but no measurable estrogenicity remained after treatment, confirming the effective mitigation of estrogen-like compounds across the treatment chain. Time-series analysis at DWTP1 and DWTP2 further confirmed stable removal performance over the four-month sampling period.

Among the six DWTPs, DWTP5 exhibited a distinct profile compared with the other plants. Notably, treated water from DWTP5 was the only sample to exhibit measurable cytotoxicity in the RTL-W1 assays, suggesting that residual bioactive compounds remained after treatment. In addition, *CYP1A* induction in treated water from DWTP5 showed only minimal reduction relative to the corresponding raw water, indicating that the treatment processes did not fully remove AhR-active contaminants. The atypical results observed for DWTP5 suggest either markedly lower removal of biologically active compounds or the formation of toxic transformation products during treatment. Unlike the other facilities, treated water from DWTP5 retained cytotoxic and *CYP1A*-inducing potential comparable to that of the raw water, indicating that the treatment steps at this plant did not sufficiently reduce the chemical stressors present in the source water. Interestingly, DWTP5 also had the lowest estrogenic activity among all raw water samples, likely reflecting characteristics of its source water. These observations underscore that even facilities with low raw-water estrogenicity may still retain other biologically active contaminants, highlighting the importance of using multiple bioassay endpoints to comprehensively assess water quality.

Although this study focused on drinking water treatment plants located in the Czech Republic, the investigated facilities represent treatment configurations commonly applied to surface water sources in many regions worldwide. All studied DWTPs employed conventional treatment steps, including coagulation, sedimentation, filtration, granular activated carbon adsorption, and disinfection, with some plants also incorporating ozonation prior to GAC filtration. At the same time, the selected facilities differed in catchment size, anthropogenic pressures, and treatment configurations, providing a range of source-water qualities and operational conditions. Consequently, the observed patterns of bioassay responses likely reflect processes relevant to many surface-water–based drinking water treatment systems beyond the studied region.

Taken together, the results suggest that conventional treatment processes—coagulation/flocculation, sedimentation, filtration, activated carbon, and disinfection—effectively eliminate estrogenic activity; however, in some cases, they may not fully remove AhR-active contaminants that induce *CYP1A*. This pattern underscores the importance of employing multiple bioassay endpoints to capture distinct classes of biological activity. While estrogenic activity was below detection in treated water, the persistent induction of *CYP1A* suggests that some polar or transformation products remain bioactive post-treatment.

This work demonstrates the value of integrating passive sampling and bioassays to assess treatment performance and the quality of residual water. *CYP1A* induction provided sensitive detection of treatment-resistant contaminants, while yeast assays confirmed effective removal of estrogenic and progestogenic activity. These findings support the use of bioassays as an effect-based monitoring framework, providing an early warning of biological activity that may persist through treatment. Future studies should link bioassay responses to specific contaminant classes using effect-directed analysis and expand time-series monitoring to evaluate long-term variability and treatment resilience under changing environmental conditions. Such efforts will be critical for safeguarding drinking water quality in the face of emerging contaminants and evolving pollution pressures.

## 5 Conclusion

This study demonstrates that integrating time-integrated passive sampling with *in vitro* bioassays provides a robust framework for evaluating the biological performance of drinking water treatment. Using POCIS extracts from raw and treated water at six Czech DWTPs, we assessed residual biological activity under realistic exposure conditions. Among the biomarkers tested, *CYP1A* gene expression was the most responsive, indicating the presence of AhR-active contaminants in raw water and, in some cases, their incomplete removal during treatment. While advanced treatment (e.g., ozonation and granular activated carbon) generally reduced *CYP1A* induction, residual activity persisted at certain plants, suggesting treatment-resistant bioactive chemicals. In contrast, *GST*, *HSP*, and *Nrf2* showed minimal responses, indicating predominantly receptor-mediated effects rather than general cytotoxicity. Estrogenic activity was consistently detected in raw waters but was below detection in all treated samples, demonstrating effective removal of estrogenic compounds. No progestogenic activity was observed. These findings underscore the value of effect-based monitoring as a complementary tool to conventional chemical analysis. Passive sampling coupled with bioassays can reveal treatment-resistant biological activity that may be overlooked by targeted approaches. Future work should focus on linking bioassay responses to specific contaminant classes through effect-directed analysis and extend monitoring across temporal and seasonal scales.

## Data Availability

The original contributions presented in the study are publicly available. The datasets generated for this study are available in the Zenodo Repository, https://doi.org/10.5281/zenodo.18107056.
