# Supplementary material for: Assessing drinking water treatment efficiency using passive sampling and cell-based bioassays
Source: Front Toxicol. 2026 Apr 23;8:1782869. doi: 10.3389/ftox.2026.1782869 (PMC13148798; doi:10.3389/ftox.2026.1782869)
Supplement: Supplementary file 1 [file DataSheet1.pdf]

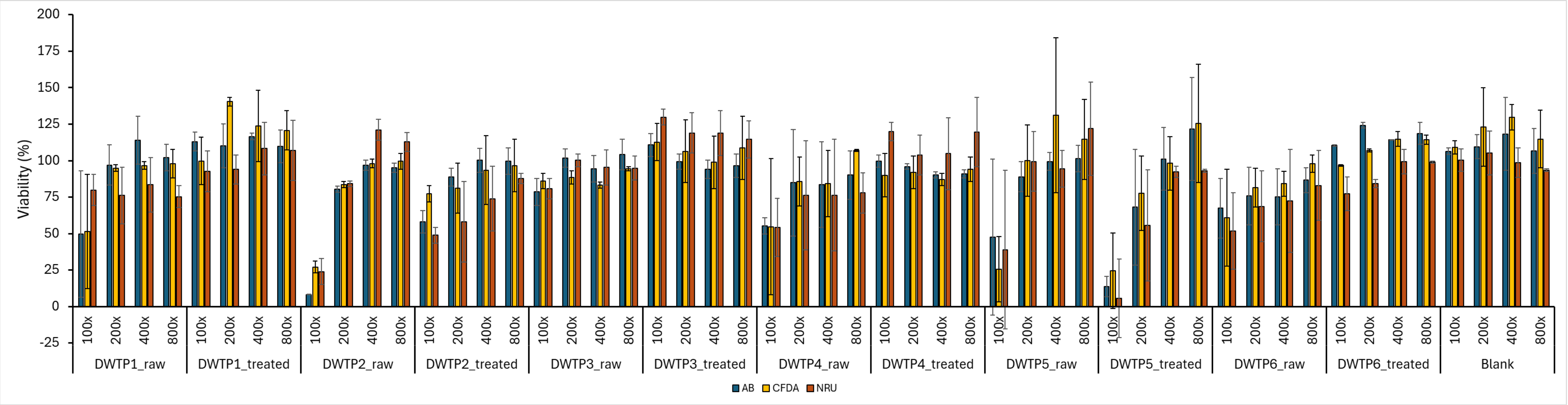

Figure S1. Viability results measured by AB, CFDA and NR assays using the RTL-W1 cell line exposed to raw and treated water from six DWTPs (DWTP1 to DWTP6). Values are means (n = 6), with error bars showing standard deviations.

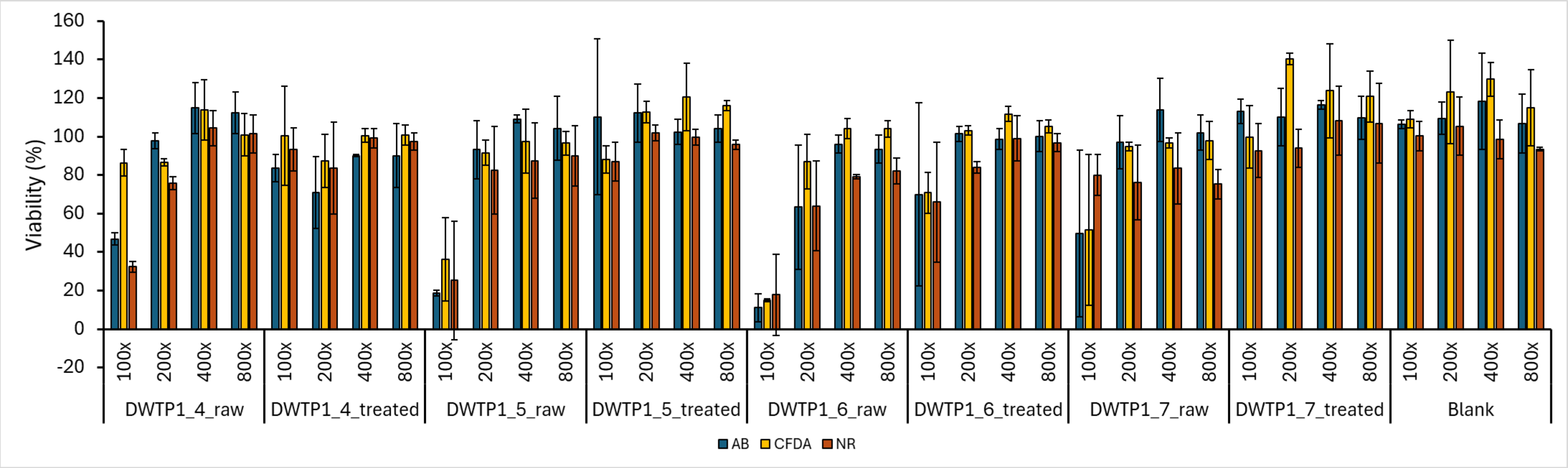

Figure S2. Viability results measured by AB, CFDA and NR assays using the RTL-W1 cell line exposed to raw and treated water from DWTP1, sampled between April and July (DWTP1_4 to DWTP1_7). Values are means (n = 6), with error bars showing standard deviations.

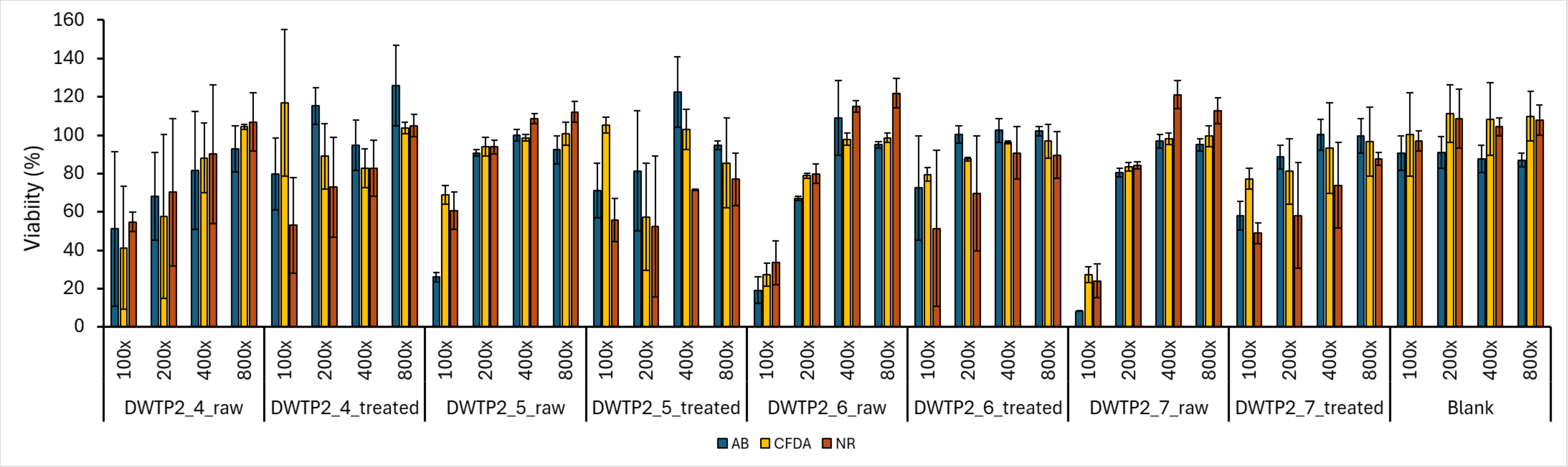

Figure S3. Viability results measured by AB, CFDA and NR assays using the RTL-W1 cell line exposed to raw and treated water from DWTP2, sampled between April and July (DWTP2_4 to DWTP2_7). Values are means (n = 6), with error bars showing standard deviations.
